# Statistical Advances in Epidemiology and Public Health

**DOI:** 10.3390/ijerph18073549

**Published:** 2021-03-29

**Authors:** Domenica Matranga, Filippa Bono, Laura Maniscalco

**Affiliations:** 1 Department of Health Promotion, Mother and Child Care, Internal Medicine and Medical Specialties, University of Palermo, 90127 Palermo, Italy; 2 Department of Economics, Business and Statistics, University of Palermo, 90128 Palermo, Italy; filippa.bono@unipa.it; 3 Department of Biomedicine, Neuroscience and Advanced Diagnostics, University of Palermo, 90127 Palermo, Italy; laura.maniscalco04@unipa.it

The key role of statistical modeling in epidemiology and public health is unquestionable. The methods and tools of biostatistics are extensively used to understand disease development, uncover the etiology, and evaluate the development of new strategies of prevention and control of the disease. Through data analysis, epidemiology can steer decision-making processes, guide health and healthcare policy, and plan and assist in the management and care of health and disease in individuals. The growing availability of large healthcare databases allows drawing new evidence in the use of healthcare interventions, drugs, and devices, and in the knowledge of population health and health inequality. Clinical decisions grounded on evidence of efficacy and safety of medical interventions can contribute to prolonging people’s lives, improving their quality, and promoting appropriateness [1].

The present Special Issue focuses on statistical methods applied to epidemiology and public health. It is advisable to enlarge the evidence-based approach to a value-based approach by including the quality and the costs dimensions from both the demand and the supply of healthcare services [2]. Thus, particular attention has also been given to the evaluation and the cost-effectiveness of procedures and services. This special issue includes twelve articles from research teams worldwide, specifically Belgium, Spain, China, Canada, Austria, and Romania, apart from Italy. These studies focus on epidemiology, public health, and health promotion [3,4,5,6], suggest appropriate statistical methodologies for specific research questions [7,8,9,10,11], and analyze the quality of services provided to patients [12,13,14].

Of the four articles in the field of public health, two studies focus on disease prevention and health promotion strategies. In detail, Matranga et al. aim to assess whether the adoption of healthy behaviors could be significantly associated with psychological well-being in a cohort of students in the healthcare area [3]. This study shows that students inclined to well-being consider healthcare professionals as models for their patients and all people in general. Furthermore, the positive relationship between a virtuous lifestyle and psychological well-being suggests the construction, development, and growth of individual skills to counteract unhealthy behaviors. The study of Fujihara et al. addresses the issue of health promotion turning to a different target [4]. Through a prospective cohort design on older people in Japan, it focuses on examining the possible association between community-level social capital and the incidence of Instrumental activities of daily living (IADL) disability. The outcomes obtained from the present investigation show that community-based interventions that are finalized to promote community-level social capital could help preventing IADL disability or reducing its incidence.

In the field of occupational epidemiology, the Special Issue includes two studies regarding job exposures, and one of these uses large databases [5]. Specifically, Maniscalco et al. investigate to what extent a change in employment contributes to cardiovascular, musculoskeletal, and neuropsychological health [5]. Using a sample of 10,530 Belgian workers in a seven-year follow-up study period, the authors find an increased risk of cardiovascular disease and a psychosocial load in association with a job change experience. The issue of students’ professional exposures has been considered by Verso et al. [6]. The authors scrutinize the prevalence of latent tuberculosis infection (LTBI) among undergraduate and postgraduate students in the healthcare area at the University of Palermo, Italy, and investigate about the occupational risk of infection among students. This study shows very few cases of LTBI, confirming that the incidence of LTBI is low among Italian students. Not only healthcare workers, but also healthcare students involved in traineeships, are daily exposed to the occupational risk of contracting tuberculosis, due to close and prolonged contact with patients. Effective prevention strategies are mandatory for University hospitals.

Regarding the articles of this Special Issue that are focused on statistical methodology, we advocate reading the study of Trivelli et al. [7] describing the spatio-temporal distribution of cardiovascular mortality, and the study of Maniscalco et al. [8] suggesting a data-driven approach to investigate the interrelationship among health indicators. These two papers give important contributions in the context of environmental epidemiology and health promotion. Specifically, the study of Trivelli et al. aims to determine the spatio-temporal association between environmental exposure to particulate matter, PM2.5 µg/m^3^, and the risk of cardiovascular mortality in the 2010–2015 period in an Italian region with a high level of air pollution and human activities, using a Bayesian smoothed approach [7]. Such an approach consists of a hierarchical mixed log-linear model with a Besag, York, and Mollié (BYM) random component in a fully Bayesian framework. As described by the authors in detail, this model produces a smoothed map of relative risks (RR) and allows extra-Poisson variability induced by the spatio-temporal data structure. The proposed model includes two Gaussian random effects, one spatially unstructured (exchangeable or white noise in the lattice) and one spatially structured (conditional autoregressive based on a Gaussian–Markov random field in the lattice). Through the smoothed maps of RRs, the authors show that the distribution of estimated risk of death for cardiovascular diseases did not change across the years between 2010 and 2015, and show evidence of three clusters of high-risk for cardiovascular diseases in Lomellina area in all the studied years.

The study of Maniscalco et al. in the field of health promotion attempts to investigate the interrelationship among statistical indicators, which are typically used to capture health multidimensionality of elderly people [8]. They are self-perceived health (SPH), quality of life (QoL) in older ages, chronic or non-communicable diseases (NCDs), global activity limitation, lifestyle, and cognitive functioning. The authors analyze data of people aged 50 or above, living in twenty-seven European countries and Israel, extracted from the Survey on Health, Ageing, and Retirement in Europe (SHARE). Through additive Bayesian network modeling, the authors consider all the indicators jointly and identify all direct and indirect relationships between them. Three directed acyclic graphs for each one of Spain, Italy, and Greece show SPH significantly associated with cognitive functioning and QoL of people aged 50 and above, and confirm the well-known association with chronic diseases. Two of the studies composing the Special Issue contribute in the field of generating real-world evidence, through a census of the available healthcare utilization databases [9] and through a study that accounts for immortal time bias in observational clinical studies [10]. These two papers supply an example of the importance of the information provided by healthcare administrative databases, which can be used in many fields of pharmacoepidemiology, such as safety, efficacy, and cost–efficacy of therapeutic strategies.

Specifically, Skrami et al. perform a census of the available Healthcare Utilization databases (HUD) across 19 Italian regions and two autonomous Trento and Bolzano provinces [9]. This paper is worthy of note, as many studies use data from HUDs to produce scientific evidence about the safety and efficacy profile of drugs. However, in order to combine the HUDs and compare diagnostic and therapeutic care pathways (PDTA) and protocols among different Italian regions, it is essential that the HUDs are harmonized and their information comparable. The work of Skrami et al. counts 352 HUDs between January 2014 and October 2016 that cover the whole population of a single region and recorded local-level data referred to the healthcare service; these databases are classified as healthcare services, conditions/diseases, and others, on the basis of the recorded observational unit. The authors find that the HUDs are homogeneous with respect to the unique personal identification code, the anonymization technique, and the DMS adopted, so that the record linkage across them is always possible. Additionally, the classification systems for diseases and drugs are found to be homogeneous across regions, while the anonymization procedures are not. This work can be considered as a model for other countries that wish to inventory their available HUDs to ease multicentric epidemiologic studies.

The study of Corrao et al. is a valuable example of real-world evidence that can be extracted from the merging of HUDs [10]. The authors aim to investigate the association between third-trimester exposure to macrolide antibiotics and the risk of preterm delivery as primary outcome and low birth weight (less than 2500 g), with smallness for gestational age and five-minutes Apgar score <7 as secondary outcomes. The merged HUDs are (1) the electronic database of the NHS beneficiaries, (2) the outpatient drugs dispensed in community pharmacies, and (3) the specialist visits and diagnostic examinations reimbursable by the NHS and the database of the Certificates of Delivery Assistance of all of Lombardy in the period between 2007 and 2017. The merging procedure gives arise to a database of 549,082 mothers with their newborns. From the methodological point of view, this paper contributes for considering the so-called immortal bias, the time window between the index date (27th gestational week) and the start of exposure (first antibiotics prescription) in which the event of interest (preterm birth) is not possible by design.

Finally, the last methodological paper of this special issue is concerned with managing data separation by logistic regression [11], which is a frequent problem in the analysis of small or sparse clinical datasets. Data can be defined as separated if there exists one covariate or a linear combination of covariates that allows a perfect prediction of some or all observations of the dataset. The authors describe several situations a medical data-analyst is faced with, as the occurrence of unbalanced outcomes [15], rare exposures, as in the case of case-control studies with controls free of any local and systemic variables [16], correlated covariates, and sparse data. The authors aim to compare Firth’s penalization, which is widely used to deal with data-separation, with the maximum likelihood method applied to an augmented dataset. Through a well-done data simulation study, they show that bringing more sampling data is not a cost-adjusted relative efficient strategy compared to logistic regression with Firth penalization.

In our Special Issue, quality evaluation of healthcare is addressed from both the patient’s point of view, in terms of quality perception of healthcare services, and from the healthcare point of view, in terms of efficiency and efficacy of care. From the patients’ viewpoint, Druică et al. investigate patients’ health services satisfaction with health services [12]. On a cross-sectional sample of 1500 Romanian patients, the authors use a partial least square–path modeling approach (PLS–PM) to determine their health services satisfaction. The authors develop a variance-based structural model, emphasizing the mediating role of trust and satisfaction with various health services categories. Results show the mediating role of trust in shaping the relationship between the procedural accuracy of health professionals, the perceived intensity of their interaction with patients, and patients’ experienced quality of the health services. As the most relevant variable for intervention, the authors detect the degree of attention patients perceive to have received. The paper suggests three methods to turn waiting time into attention deserved to patients.

Gálvez et al. evaluate hospitals’ efficiency by comparing a new model of organizational innovation based on Advanced Practice Nurse in the care of people with Ostomies (APN-O) versus usual care [13]. The study involves twelve Andalusian hospitals that implement this model. On a total of 75 patients followed-up for six months, the authors analyze clinical outcomes, healthcare resources, health-related quality of life, and willingness to pay. The economic evaluation takes into consideration the healthcare direct and indirect cost and finds evidence of an increased value-based healthcare in ostomies. This study suggests that APN-O is an effective and highly efficient patient management model for improving patients’ health status.

Li et al. deal with different objectives in the efficiency evaluation of Chinese hospitals [14]. In a first step, efficiency and change in efficiency are evaluated using Data Envelopment Analyses and the Malmquist index. The study considers 29 provinces, where data from 1336 hospitals are observed in a time-span from 2003 to 2016; in a second step of the study, starting from the efficiency differences calculated in the first step of the analysis, the authors use the Theil index to obtain the efficiency decomposition. Then, they find the inefficiency determinants through the Grey correlation analysis. The study results show that the township hospitals achieve efficiency gains in most provinces and that the intra-regional difference is the major cause of the overall efficiency scores’ difference. This paper further examines the Grey correlations between overall provincial efficiency difference of Chinese township hospitals and the determinants of such a difference, within each of the eastern, central, and western regions of China.

In conclusion, the authors suggest that local governments should take measures to improve the level of education, increase public financial support for township hospitals, and guide household expenditure to invest more on health care and medical services through public education, so as to shrink the differences among provinces. Furthermore, township hospitals in relatively backward provinces should not ignore the effects of increasing the proportion of licensed doctors and assistant doctors, and the proportion of managerial personnel in the total number of medical personnel.

The Special Issue encompasses different and valuable works related to what extent statistical disciplines are important in the fields of epidemiology, public health, and health promotion. The selected studies for this Special Issue contribute relevant information that may help suggesting appropriate statistical methodologies for specific biomedical research questions and analyzing the quality of services provided in public health. Furthermore, it highlights several statistical methods currently used in public health and epidemiological studies and some of the frequent problems encountered in the analysis of clinical datasets.

## Data Availability

No new data were created or analyzed in this study. Data sharing is not applicable to this article.
